# Characterization and phylogenetic analysis of the complete mitochondrial genome of *Sinocyclocheilus wenshanensis* (Cypriniformes: Cyprinidae)

**DOI:** 10.1080/23802359.2022.2054383

**Published:** 2022-04-04

**Authors:** Chunqing Li, Fang Hu, Junxian He, Xutong Li, Hongfu Yang, Weixian Li, Shanyuan Chen, Heng Xiao

**Affiliations:** a School of Ecology and Environmental Science, Yunnan University, Kunming, China; b School of Life Sciences, Yunnan University, Kunming, China; c Fisheries Administration of Qiubei County, Wenshan, China; d Heilongtan Reservoir of Shilin County, Kunming, China

**Keywords:** *Sinocyclocheilus wenshanensis*, mitochondrial genome, phylogenetic relationship

## Abstract

*Sinocyclocheilus wenshanensis* is a cyprinid fish species endemic to Southwestern China. In this study, we first sequenced and characterized the complete mitochondrial genome (mitogenome) of *S. wenshanensis* by next-generation sequencing method. The entire length of mitogenome is 16,595 base pairs (bp), containing 13 protein-coding genes, two ribosomal RNA genes, 22 transfer RNA genes, and a control region. Its gene arrangement pattern was identical to other previously reported *Sinocyclocheilus* fishes. The overall base composition is 31.12% A, 16.63% G, 25.45% T, and 26.80% C, with AT content of 56.57%. Phylogenetic analysis using mitogenome of 26 Cyprinidae fishes showed that *S. wenshanensis* are closely related to *S. aluensis* and *S. oxycephalus*. This work would provide molecular information fundamental to future phylogenetic analyses among *Sinocyclocheilus* species.

*Sinocyclocheilus wenshanensis* belonging to the genus *Sinocyclocheilus* (Cypriniformes, Cyprinidae) is a freshwater fish species that distributes only in the karst landform areas within Wenshan, belonging to Red River drainage, located in Southeast Yunnan, China (Yang et al. 2017). In the present study, we first sequenced and characterized the complete mitochondrial genome (mitogenome) of *S. wenshanensis* by next-generation sequencing method, which would facilitate future studies on *Sinocyclocheilus* phylogenetics.

The specimen of *S. wenshanensis* under study was collected in Wujiazhai (23.23°N, 104.05°E), Xigu Town, Wenshan, Yunnan Province, China. The specimen was preserved in 95% ethanol and deposited in the Zoological Specimen Museum of Yunnan University (Chunqing Li, lichq@ynu.edu.cn) under the voucher number YNUSW20160703016. Genomic DNA from muscle tissue was extracted by DNeasy Blood & Tissue Kit (QiaGen, Hilden, Germany). The DNA library was prepared following Illumina’s instruction and sequenced on Illumina Miseq (Illumina, San Diego, CA). It generated 3,506,072 clean reads with 699,718,556 base pairs (bp) in total. The complete mitogenome sequence of *S. wenshanensis* was assembled with A5-miseq v20150522 (Coil et al. 2015) and SPAdes (Bankevich et al. 2012). Assembled mitogenome sequence was annotated using the MitoAnnotator on the MitoFish homepage (Iwasaki et al. 2013). All transfer RNA (tRNA) genes were identified by MITOS (Bernt et al. 2013) and tRNAscan-SE search server (Lowe and Chan 2016). The mitogenome Circular map was drawn by the MitoAnnotator on the MitoFish homepage (Iwasaki et al. 2013).

The complete mitogenome of *S. wenshanensis* had been deposited in GenBank database (accession number OM001088). The mitogenome sequence has 16,595 bp in length, consisting of 13 protein-coding genes (PCGs), 22 tRNA genes, two ribosomal RNA (rRNA) genes, and one control region. The gene organization and structure were consistent with those of previously reported mitogenomes of *Sinocyclocheilus jii* (Li et al. 2017), *Sinocyclocheilus multipunctatus* (Zhang and Wang 2018), and *Sinocyclocheilus tingi* (Li et al. 2019). The overall base composition is 31.12% A, 16.63% G, 25.45% T, and 26.80% C, with AT content of 56.57%. Most mitochondrial genes were encoded on the heavy strand (H-strand), except that eight tRNA genes and the ND6 gene were encoded on the light strand (L-strand). All PCGs have start codon ATG except for COI with start codon GTG. Six PCGs have complete stop codons TAG (ND1) and TAA (COI, ATP8, ND4L, ND5, and ND6). The ATP6 and COIII genes use incomplete stop codon (TA–). The ND2, COII, ND3, ND4, and CYTB genes end with incomplete stop codon (T–). The lengths of 22 tRNA genes range from 67 to 76 bp, which can be folded into a typical cloverleaf structure. The lengths of 12S and 16S rRNA genes were 954 and 1680 bp, respectively. The control region (D-loop) is 941 bp in size, located between tRNA-Pro and tRNA-Phe genes.

To determine the phylogenetic position of *S. wenshanensis*, phylogenetic trees were reconstructed using MrBayes v3.2.5 (Ronquist et al. 2012). The mitogenome of *S. wenshanensis*, together with 23 *Sinocyclocheilus* mitogenomes and two outgroup mitogenomes (*Barbodes binotatus* and *Poropuntius huangchuchieni*) available in GenBank database were used to perform phylogenetic analysis. The phylogenetic results (Figure 1) revealed that *S. wenshanensis* was closely related to *Sinocyclocheilus aluensis* (Cui et al. 2021) and *Sinocyclocheilus oxycephalus* (Li et al. 2018), and that 24 *Sinocyclocheilus* species were grouped as a monophyletic clade with strong supports. Phylogenetic analysis reveals *Sinocyclocheilus* form a solid monophyletic group, which was consistent with the traditional morphological classification and previous phylogeny studies based on partial mitochondrial genes or complete mitogenomes (Xiao et al. 2005; Zhao and Zhang 2009; Yang et al. 2021).

**Figure 1.**
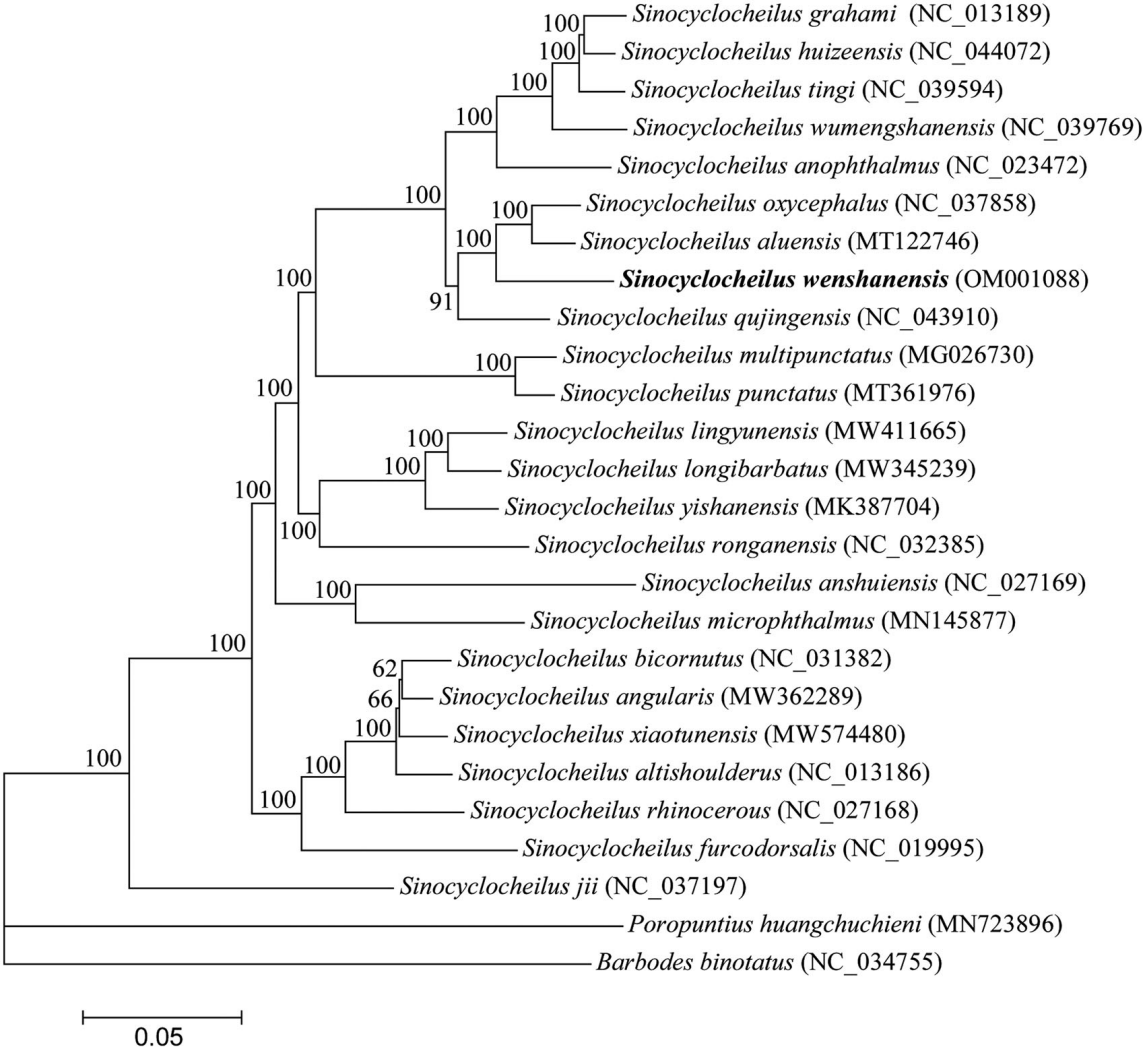
Phylogenetic tree of 24 *Sinocyclocheilus* fishes and two outgroups based on complete mitogenome sequences. The accession numbers for each species are indicated in parenthesis. The nodal numbers represent the posterior probabilities for Bayesian analysis.

## Authors contributions

Chunqing Li: analysis and interpretation of the data; investigation; drafting of the paper. Fang Hu: analysis and interpretation of the data; investigation. Junxian He: analysis and interpretation of the data; investigation. Xutong Li: formal analysis; methodology. Weixian Li: taxonomic identification. Hongfu Yang: sample collection. Shanyuan Chen: conception and design; funding acquisition; supervision; revising the paper critically for intellectual content. Heng Xiao: conception and design; funding acquisition; supervision; reviewing the final approval of the version to be published. All authors agree to be accountable for all aspects of the work.

## Data Availability

The genome sequence data that support the findings of this study are openly available in GenBank of NCBI at https://www.ncbi.nlm.nih.gov under the accession no. OM001088. The associated BioProject, SRA, and Bio-Sample numbers are PRJNA796176, SRR17552844, and SAMN24817860, respectively.
